# Collaborative Development of an Instrument to Monitor Physical Activity Promotion Based on Policy-Makers’ Needs – the TARGET:PA Tool

**DOI:** 10.34172/ijhpm.8720

**Published:** 2025-09-23

**Authors:** Peter Gelius, Sven Messing, Karim Abu-Omar, Isabel Marzi, Franziska Beck, Wolfgang Geidl, Eva Grüne, Antonina Tcymbal, Anne Kerstin Reimers, Klaus Pfeifer

**Affiliations:** ^1^Institute of Sport Sciences, Université de Lausanne, Lausanne, Switzerland.; ^2^Department of Sport Science and Sport, Friedrich-Alexander-Universität Erlangen-Nürnberg, Erlangen, Germany.

**Keywords:** Sports, Health, Active Transport, Tool Development, Policy

## Abstract

**Background:**

To support policy development, a number of tools are available to inform policy-makers about the current status of physical activity (PA) promotion in a specific country. However, a recent exchange between policy-makers and researchers in Germany revealed two major gaps: First, examples of successful good practice projects are often not selected in an objective and systematic process. Second, there is usually no systematic assessment of "routine practice," ie, PA promotion activities already taking place on large scale and regular basis. These issues are addressed by the newly developed TARGET:PA tool.

**Methods:**

The TARGET:PA tool was developed in a co-production process involving researchers from the World Health Organization Collaborating Centre for Physical Activity and Public Health (WHO CC) at FAU Erlangen-Nürnberg and the policy unit in charge of cardiovascular diseases, diabetes and non-communicable diseases at the German Ministry of Health. We documented the development process, details on the structure of the tool itself, and the outputs produced using the tool.

**Results:**

The development process involved a negotiation process between researchers and policy-makers and the need to adapt to extended decision-making timelines within the ministry. With regard to PA behavior at the individual level, the TARGET:PA tool includes an overview about (1) PA recommendations and (2) national PA prevalence rates. At the organizational/policy level, it contains information on (3) recommendations for PA promotion, (4) routine practice, (5) good practice projects, and (6) policies. Key outputs of the tool are policy briefs as well as scientific background documents.

**Conclusion:**

The TARGET:PA tool provides added value as it can support the integration of "good" and "routine" practices into the monitoring of PA promotion. While the tool has been developed and tested in Germany, it has the potential to be adapted to other countries, either by directly utilizing the tool or by emulating the collaborative development process to design new instruments adapted to specific contexts.

## Background

**Key Messages**

**Implications for policy makers**

- The TARGET:PA tool helps provide a systematic overview of a country’s physical activity (PA) promotion landscape. By systematically identifying good practice, existing programs, and implementation gaps, it can support governments in developing future policies.
- Tool development was based on a co-production process involving government officials and scientists, focusing on the monitoring needs of the government. As a consequence, the tool follows a different, less “controversial” theory of change than overviews produced by independent researchers.
- The tool has the potential to be turned into a regular monitoring system and to be adapted to other countries and/or target groups. This may either be achieved through a direct transfer of the tool or by replicating the co-production process to develop a new instrument tailored to the specific context.

**Implications for the public**

It is crucial for governments to understand the status quo of physical activity (PA) promotion in their country. A comprehensive overview of PA promotion practices—including good practice (projects with demonstrated effectiveness) and routine practice (large-scale regular programs already in place)—can be a valuable foundation for policy development. TARGET:PA is a tool that was co-created by researchers and policy-makers to produce short overview on PA promotion (policy briefs) that may inform the public and future policy development. This could contribute to healthier environments that promote PA and enhance population health.

Physical activity (PA) is associated with numerous health benefits, such as improved cardiorespiratory fitness,^1,2^ the primary and secondary prevention of several chronic diseases, and reduced premature mortality.^3^ Studies have shown that there is a dose-response relationship between PA and health outcomes,^3^ ie, even small increases in population PA levels can have a significant public health benefit. In order to increase the global prevalence of PA, the World Health Organization (WHO) has adopted a Global Action Plan on PA that aims to advise policy-makers on how to promote health-enhancing PA.^4^ For member states of the European Union (EU), the Council Recommendation on Promoting Health-Enhancing PA across Sectors is a key policy at supranational level.^5^ Around the world, many countries have developed national PA policies,^6^ and there is evidence on the effectiveness of such policies to promote PA.^7^ But in spite of these efforts, global data show that it is unlikely that WHO’s PA target of a 10% relative reduction in insufficient PA will be met without further action.^8^ Consequently, researchers highlight the urgent need to prioritize and scale up policies to increase population levels of PA.^8^

 To support policy development, a number of tools are available to inform policy-makers about the current status of PA promotion in a specific country.^9,10^ For instance, the implementation of the EU Council Recommendation is monitored by the European Commission and the WHO Regional Office for Europe on a triennial basis, using a monitoring framework that helps member state governments to collect country-specific information on 23 indicators across 10 thematic areas.^11^ WHO’s Health-Enhancing PA Policy Audit Tool (HEPA PAT)^12^ can be used by intersectoral stakeholder alliances to complete 29 open-ended questions on national PA promotion in a collaborative process. The newly developed PA-Environment Policy Index^13^ focuses on benchmarking policy implementation: A coalition of researchers, experts and policy-makers collects information on existing national policies and programs and subsequently compares their design and level of implementation against 45 good practice statements as well as international exemplars of best practice. Other well-known examples of monitoring tools are the Report Cards of the Global Observatory for PA^14^ and the Active Healthy Kids Global Alliance, both of which are compiled by PA researchers but sometimes involve limited participation from national policy-makers.^15^

 Each of these tools is based on certain, partly implicit theories of change, ie, assumptions on how impact on policy-making is generated^9^: For example, instruments that are intended “to stimulate critical debate, greater awareness, a broader dialogue among relevant actors and a higher sense of ownership within countries”^16^ (like the HEPA PAT) tend to employ co-production approaches that can “produce research findings that are more likely (…) relevant to and used by the end users.”^17^ By contrast, research-driven approaches (like the Global Observatory and Active Healthy Kids report cards) may aim to impact policy-making by publicly pointing out major policy gaps, sometimes even using school grades.^9,18^ The output formats of these tools vary as well: While some of them aim to provide exhaustive information on the national policy landscape in the form of detailed reports (eg, HEPA PAT), others use extremely condensed formats to convey their main findings to policy-makers (eg, the report cards of the Global Observatory for PA and the Active Healthy Kids Global Alliance).

 Most existing tools do an excellent job at covering a broad range of aspects in the field of PA promotion, notably PA prevalence rates in different population groups, select social and environmental determinants of PA, national-level PA policies in different sectors, and central political actors involved in PA policy-making. However, existing tools may not always reflect the specific information needs of policy-makers during the development process of new PA policies, and governments may request data that necessitate the development of tailored PA promotion monitoring tools. In the case reported in this paper, exchanges between policy-makers and researchers revealed that important information requested by the government was unavailable and could not be obtained using existing instruments. In particular, this pertained to evidence-based pilot projects with a high chance for nationwide scale-up (“good practice”) and to existing large-scale projects and programs that have existed for a long time (“routine practice”), eg, PA offers by sport organizations, regular outdoor time in childcare facilities, or PA counselling based on green prescriptions schemes. While information on good practice may help governments make important decisions for future project funding, routine practice is particularly relevant as it already has a high reach, and substantial public health impact might be generated by further optimizing existing programs instead of introducing new ones from scratch.

 To address the government’s information needs, researchers used a collaborative process to develop TARGET:PA, a new tool that addresses the above-mentioned issues by emphasizing the systematic assessment of both good practice projects and routine practice. This article describes the development process of the tool and its main components. It shows how the tool can be applied to investigate PA promotion in a particular country and discusses how our experience may be useful to other countries, either by directly using the TARGET:PA tool or by imitating the collaborative process to develop their own tools.

## Methods

 The TARGET:PA tool was developed in a co-production process involving two parties: Academia was represented by the WHO Collaborating Centre for Physical Activity and Public Health (WHO CC) at FAU Erlangen-Nürnberg, Germany. WHO Collaborating Centres are independent scientific institutions that conduct research on behalf of the WHO to support the organization’s policy-making efforts. WHO CCs are often supported financially by their national governments and also serve as national centers of competence in their respective field.

 The other actor was the policy unit in charge of cardiovascular diseases, diabetes and non-communicable diseases at the German Ministry of Health. The Ministry of Health initiated the exchange by expressing the need for a comprehensive overview of the current status of PA promotion for specific target groups in Germany. The request needs to be seen in the context of the updated German national action plan on PA and nutrition “IN FORM,” which focuses on vulnerable target groups (eg, first 1000 days, children, older adults)^19^: The ministry required an overview of the current situation to form a basis for its future policy action in these fields. However, such a synopsis was not readily available due to the complex distribution of competences across different political sectors and levels within Germany’s federal system of government.

 We documented the collaborative development process of the new PA promotion monitoring tool using meeting minutes of video conferences between the two teams, notes from phone calls, and a review of e-mail exchanges between meetings. Data were compiled into a timeline and a narrative description of the process. Following the finalization of the tool and its approval by the ministry, researchers compiled a series of policy briefs for different age and target groups, which were published in long and short form by the Ministry of Health. We conducted a brief analysis of both the tool (TARGET:PA) and the resulting policy brief to summarize their main features. The purpose of this exercise was to present the experience with a collaborative tool development process for PA promotion monitoring as well as its outputs from the perspective of the participating researchers. As a consequence, the results are necessarily subjective in nature and have not been checked for data gaps or potential bias.

## Results

###  Collaborative Tool Development Process

**Figure 1:**
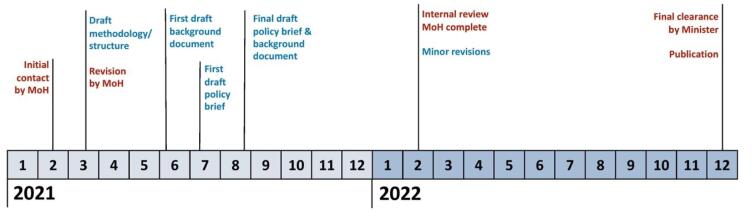
Development Timeline of the First Policy Brief Based on the TARGET:PA Tool. Abbreviations: PA, physical activity; MoH, Ministry of Health.

Figure 1 provides an overview of the process of developing the TARGET:PA tool and completing the first in a series of four policy briefs for different target groups. The initial contact was made by the Ministry of Health in February 2021, requesting the development of policy briefs for its future policy development efforts in the field of PA and health. The ministry emphasized its priority of integrating both good and routine practice systematically into the data collection process. In a series of meetings during the subsequent weeks, ministry officials and the WHO CC leadership negotiated the details of the upcoming work, including the timeline, the need for a dedicated tool, and the length of the final output format. It was decided to try to prepare the final draft for a first policy brief on PA promotion for children and adolescents in Germany within six months.^20,21^ As none of the existing monitoring tools covered the requested aspects in sufficient detail, the WHO CC decided to develop a new tool to facilitate data collection. Due to the needs of the ministry, the tool was required to be comprehensive, adaptable, target-group specific, and applicable in a rapid process. In addition, it was decided that two output documents would be produced: A short version of under ten pages (the “policy brief”) and a long version without a page limit (the “background document”). Together, these two documents would strike a balance between the needs of policy-makers for concise information and the need of academics for scientific rigor.

 As a next step, the WHO CC established an ad hoc research group that included ten researchers with expertise in the areas of PA prevalence rates, interventions, and policies. Within the next weeks, researchers developed a draft version of the methodology and structure (the first draft version of the TARGET:PA tool), which was subsequently reviewed and slightly modified in several conference calls of the ministry staff and the research group. By June, the WHO CC submitted a first draft of the background document summarizing the data collected using the tool, and a first draft of the policy briefs about five weeks later. Following feedback from ministry officials, the final drafts of both documents were officially submitted to the ministry at the end of August 2021. In February 2022, the ministry completed its in-house review of the two documents and requested minor revisions from the WHO CC, which were provided later that month. The final clearance from the German Minister of Health was obtained by December 2022, and both the policy brief and the background document were published under the auspices of the ministry. A detailed report on the two documents, including data collection, data analysis, and main results, has been published elsewhere.^22^ In the context of a follow-up project^23^ awarded by the ministry for the development of three more policy briefs for additional target groups, the WHO CC critically reviewed the methodology of data collection, resulting in a finalized version of the TARGET:PA tool.

###  Characteristics of the TARGET:PA Tool

**Figure 2:**
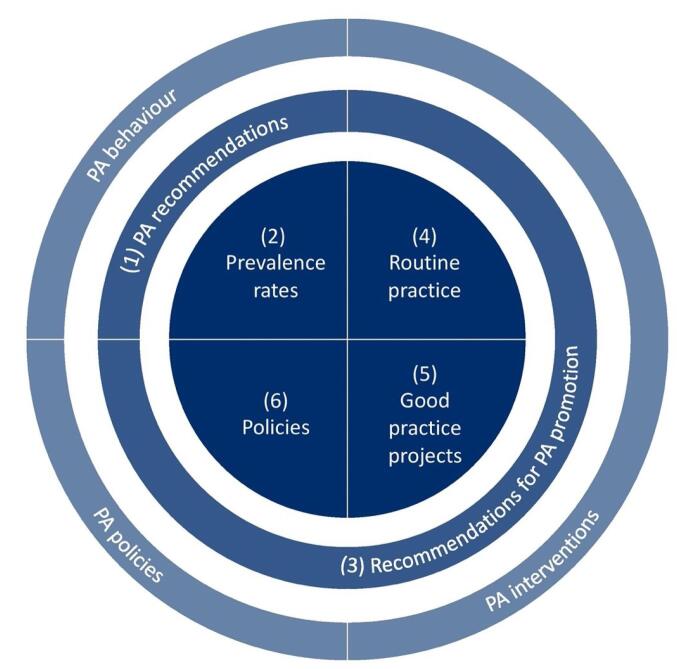
Structure of the TARGET:PA Tool. Abbreviation: PA, physical activity.

 From a theoretical perspective, the development of the TARGET:PA tool was based on a typology of three types of scientific evidence related to PA behavior/prevalence rates (type I), PA interventions (type II), and PA policies (ie, the framework in which interventions are implemented, type III).^7,24^ Furthermore, the TARGET:PA tool is aligned with two groups of recommendations. The first group are PA recommendations that are targeted at individuals and represent an evidence-based, clinically guided framework that centers on the nature, duration, intensity, and volume of PA.^25^ The second group are recommendations for PA promotion that target governments and stakeholders in different sectors and at different levels, and that refer to interventions and policies for PA promotion during daily living or in specific settings.^25^ As shown in Figure 2, the TARGET:PA tool aims to address the different types of scientific evidence and recommendations by focusing on (1) PA recommendations and (2) national PA prevalence rates, (3) recommendations for PA promotion, and data on (4) routine practice, (5) good practice projects, and (6) policies within a particular country. While the first two elements refer to PA behavior, the other elements refer to measures for PA promotion.

 The six elements of the TARGET:PA tool can answer key questions of relevance for decision-makers in the field of PA promotion (Table 1).

**Table 1:** Key Questions and Suggested Methodology

**Section**	**Key Questions**	**Suggested Methodology**
**Physical Activity Behavior**		
(1) PA recommendations	(a) What are the PA recommendations for this target group? (b) If there are national PA recommendations, how do they compare with international recommendations? (c) If there are no national PA recommendations, what can we learn from international recommendations or studies about the volume and type of PA recommended for this target group?	Narrative synthesis of international (and national) PA recommendations
(2) PA prevalence rates	(a) What are the PA prevalence rates of the target group and what share fulfills current PA recommendations? (b) Are there any differences in PA prevalence rates and/or adherence to PA recommendations, eg, based on age, gender, socio-economic status, education, or other relevant indicators? How did the COVID-19 pandemic impact PA prevalence rates and/or adherence to PA recommendations? (c) Are there any gaps in the available data on target group specific PA prevalence rates?	(a) Identification of scientific publications on target group specific PA prevalence rates and adherence to PA recommendations (b) Data extraction and analysis with a focus on aspects of interest (eg, differences by age, gender and/or socioeconomic status, influence of COVID-19 lockdowns) (c) Visualization of data availability
**Physical Activity Promotion**		
(3) Recommendations for PA promotion	What are recommendations for PA promotion for this target group?	(a) Narrative synthesis of international (and national) recommendations for PA promotion (b) Identification of relevant sectors/settings for PA promotion for this target group
(4) Routine practice	(a) What is the routine practice for PA promotion for this target group in the country (activities that take place on a regular basis, eg, due to legal regulations, funding mechanisms, or the initiative of organizations)? (b) What is the reach, durability, and effectiveness of this routine practice?	Semi-structured interviews or an online survey with experts from different sectors/settings
(5) Good practice projects	(a) Which projects are examples of good practice in PA promotion for this target group in the country (evidence-based projects with proven effectiveness)? (b) What is their reach and durability, and which effects were proven?	(a) Identification of projects in relevant databases (b) Selection of good practice projects based on quality criteria (c) Structuring good practice projects based on sector/setting and assessment of their quality
(6) Policies	What policies exist to promote PA among the target group in the country?	(a) Data collection via established tools for PA policy monitoring (if available) and additional desk research (b) Identification of key policy documents and key stakeholders for all relevant sectors/settings

Abbreviation: PA, physical activity.

####  (1) Physical Activity Recommendations

 A synopsis of PA recommendations for the target group serves as a guideline for policy-makers to interpret country specific PA prevalence rates. This includes current international recommendations from WHO^26,27^ as well as national recommendations adopted in the investigated country (eg, for Germany).^28^ In addition, it seems helpful to analyze previous WHO recommendations^29^ if they were used in country specific studies to identify the adherence to PA recommendations.

####  (2) Physical Activity Prevalence Rates

 A systematic search for country specific scientific publications is the basis to identify PA prevalence rates of the target group. In order to identify gaps in the data (eg, regarding prevalences of PA for specific age groups), relevant study characteristics such as sample size and confidence intervals of each study can be identified and compared. This matrix provides a visual overview of data availability.

 A secondary data analysis of PA prevalence rates informs policy-makers about the PA behavior of the target group. This analysis provides information on the adherence to PA recommendations. In this context, population PA levels of vulnerable sub-groups should be a special focus. Based on data availability, this can include analyses based on age, gender, socioeconomic status, education, disabilities, or other relevant indicators.

####  (3) Recommendations for Physical Activity Promotion

 A synopsis of target group specific recommendations for PA promotion guides the interpretation of routine practice, good practice, as well as policies. The synopsis may be based on current recommendations by WHO,^4^ WHO/Europe,^30^ the Council of the EU,^5^ and international expert associations such as the International Society for Physical Activity and Health.^31^ If applicable, national recommendations for PA promotion can be included as well.^28^

 The recommendations can be structured into different categories, which are derived from existing international, national and scientific recommendations for PA promotion^22^ and based on the targeted sectors and/or settings (Table 2). These categories help to collect and present data in the second part of the TARGET:PA tool, PA promotion, in a comparable manner.

**Table 2:** Suggested Categories for Applying the TARGET:PA Tool

**Children and Adolescents**	**Adults**	**Older Adults**
**General Categories**		
Family & home setting	[Family & home setting]	Family & home setting
Sport	Sport	Sport
Health	Health	Health
Traffic	Traffic	Traffic
Urban planning	Urban planning	Urban planning
Community	Community	Community
[Mass media campaigns]	Mass media campaigns	[Mass media campaigns]
Other	Other	Other
**Target Group Specific Categories**		
Childcare facilities	Worksite	Senior residencies
Schools	-	-

Abbreviation: PA, physical activity.

####  (4) Routine Practice 

 For each of these categories, routine practice for PA promotion should be identified, eg, via desk research, semi-structured expert interviews and/or an online survey. In this context, it is important to understand the substantial difference between good practice projects (eg, pilot projects run by academic institutions, with proven effectiveness) and routine practice that takes place on a regular basis (eg, programs run by governmental or civil society organizations such as municipalities, schools, sport federations, or cycling federations). In contrast to good practice projects, routine practice is often not documented systematically in databases or scientific publications.

 As part of this process, the efficacy, reach, and maintenance of routine practice can be identified based on a streamlined set of quality criteria. For TARGET:PA, we used a set that had been previously developed based on the RE-AIM framework^32^ and quality criteria for PA promotion interventions^33^:

- Reach: How many people have been reached in total by the routine practice/project/intervention?
- Effectiveness: Is there evidence for the effectiveness of the routine practice/project/intervention?
- Maintenance: For how many years did/has the routine practice/project/intervention run?

####  (5) Good Practice Projects

 Good practice projects can be identified utilizing national databases that include projects for PA promotion. Relevant databases might be available in the health and/or sport sector,^34,35^ and include hundreds of projects for PA promotion for a particular target group.^22^ From these databases, data on projects targeting PA can be extracted and categorized according to the previously developed categories.

 The assessment of projects can be based on the streamlined set of quality criteria (reach, effectiveness, and maintenance). As effectiveness seems to be a core criterion to identify evidence-based good practice projects, all projects that do not fulfill this criterion may be excluded. For the included good practice projects, efficacy, reach, and maintenance should be assessed and described. If the information provided by national databases does not address all these criteria, additional data can be collected via desk research from project reports or websites.

####  (6) Policies

 With regards to public policies, it is of particular importance to identify key policy documents as well as key governmental and non-governmental organizations. These data can be collected through desk research, a review of the scientific literature, or a combination of both. To achieve this, information from existing tools for policy monitoring can be used, such as the EU/WHO Physical Activity Country Factsheets,^11^ the HEPA PAT,^12^ the Physical Activity Environment Policy Index,^13^ and the Global Observatory for PA or Active Healthy Kids Report Cards.^14,36^

 A content analysis of key policy documents may help to extract policies that are relevant for the investigated target group. This information can be structured according to the previously developed categories to facilitate the identification of policies for specific sectors or settings.

 Similarly, the identified governmental and non-governmental organizations can be structured based on these categories.

###  Applying the TARGET:PA tool

 As summarized in Table 1, the TARGET:PA tool is based on a variety of methods such as narrative syntheses, quantitative data analysis, semi-structured expert interviews or an online survey, and a systematic assessment of projects from national databases. However, the methodology may be adapted – ideally in a co-production process with government officials – when the tool is applied in different countries or for different target groups.

###  Output: Policy Brief and Background Document

 The main output of the TARGET:PA tool is a policy brief. It includes key results, key policy recommendations, and cross-references to a scientific background document. The policy brief is developed based on scientific evidence and finalized in close collaboration with government staff. This process of co-production ensures that the content is relevant for decision-makers inside and outside government, and may result in a formal endorsement of the policy brief by the ministry/government itself.

 The scientific background document includes a detailed description of the methodology and a presentation of all results. In addition, it may include conclusions for each section, and a reflection of the methodological limitations.

 The suggested structure for both documents is presented in Table 3. In Germany, the first policy brief on PA and PA promotion for children and adolescents was published in 2022, comprising nine pages.^22,37^ The accompanying detailed scientific background document had an overall length of 62 pages.^38^ In the context of the follow-up project awarded to the WHO CC by the Ministry of Health, three additional sets of policy briefs and background documents were prepared: one on adults (aged 18–65), older adults (over 65), and adults with non-communicable diseases.^39^

**Table 3:** Level of Detail of Background Document and Policy Brief (Based on the First Case Study for Children and Adolescents in Germany)

**Section**	**Policy Brief**	**Background Document**
**PA**		
(1) PA recommendations	Brief summary of PA recommendations	Synopsis of international and national PA recommendations
(2) PA prevalence rates	(1) Availability of data across age groups and PA behaviors (2) Brief summary of PA prevalence rates across age groups, including gender differences	(a) Availability of data across age groups and PA behaviors (b) PA prevalence rates across age groups (c) Differences by gender and socioeconomic status (d) Influence of COVID-19 containment measures on PA behaviors
**PA Promotion**		
(3) Recommendations for PA promotion	Brief summary of recommendations for PA promotion in different settings and sectors	Synopsis of international and national recommendations for PA promotion in different settings and sectors
(4) Routine practice	Brief presentation of routine practice within each setting/sector	(a) Detailed presentation of routine practice within each setting/sector, and information on their estimated efficacy, reach, and maintenance (b) Presentation of key messages of interviewed experts
(5) Good practice projects	Brief presentation of number of projects identified within each setting/sector and the good practice projects	Detailed presentation of identified good practice projects within each setting/sector, their key features, and information on their efficacy, reach, and maintenance
(6) Policies	Brief presentation of key policy documents and the targeted settings/sectors Brief presentation of key stakeholders for PA promotion for each setting/sector	(a) Detailed presentation of key policy documents and their target group specific content (b) Brief presentation of key stakeholders for PA promotion for each setting/sector (c) Presentation of greatest progress and biggest challenges within each setting/sector (d) Reference to other monitoring tools for PA and PA promotion

Abbreviation: PA, physical activity.

## Discussion

 TARGET:PA is a tool that provides potential added value for the field of PA promotion monitoring, as existing tools only partially reflect the needs of policy-makers for a systematic and objective monitoring of good practice projects and routine practice. Furthermore, the tool is comprehensive, adaptable and target group specific, and can be applied in a rapid process, if needed. These aspects, combined with the needs of the German Federal Ministry of Health, called for co-producing a new tool. With regard to PA behavior at the individual level, the TARGET:PA tool includes an overview about (I) PA recommendations and (II) national PA prevalence rates. At the organizational/policy level, it contains information on (III) recommendations for PA promotion, (IV) routine practice, (V) good practice projects, and (VI) policies. While the related policy brief focuses on key results and key policy recommendations, it is supplemented by a scientific background document that provides additional information concerning the methodology as well as a presentation of all results.

 The development process can be classified as an example of a “policy pull,”^40,41^ with a political actor demanding a specific research to be produced and tailored to its own policy needs. At the start of the process, compromises had to be negotiated between researchers and policy-makers. For example, different ideas regarding the output format (concise information for politicians vs. scientifically sound reporting) led to the development of two different documents rather than a single one (as originally planned). The timeline from initial contact to final publication indicates that government institutions can have long response times due to their internal decision-making structures, requiring flexibility from researchers. The process of developing and applying TARGET:PA may be unique to the German context, but it may still hold important lessons for researchers and policy-makers in other countries aiming to collaborate on PA promotion monitoring. However, applying the tool in other countries might not necessarily be based on a “policy pull” process,^40,41^ ie, different approaches might be used to ensure government involvement.

 The final form and shape of TARGET:PA is based on specific demands made by the German Ministry of Health. However, several of these features may have an added value well beyond the German context: First, its focus on monitoring routine practice for PA promotion is unique compared to existing tools and aligned with the systematic identification of good practice projects based on theory-based and objective quality criteria.^32,33^ This allows it to identify strengths and weaknesses of routine practice and good practice projects regarding their public health impact (efficacy, reach, and maintenance). Based on this information, policy-makers are enabled to develop targeted policies and strategies to increase the public health impact of ongoing activities – either by scaling up effective good practice projects or by further developing activities that already take place on a regular basis (routine practice).

 Second, the identification of gaps is an inherent part of the process. The mapping of available data on PA prevalence rates can identify research gaps for specific age groups or PA behaviors (eg, organized sport, active transport). Gaps might also be identified in the context of routine practice, good practice projects, and policies, eg, for specific sectors or settings. With this information, policy-makers can develop strategies to address gaps in data availability and PA promotion efforts.

 A third aspect is that the policy brief can have an impact on policy-making. This is facilitated by a “policy pull” process^40,41^ and supported by a co-production approach of government officials and scientists. The co-production built trust and ensured communication at every stage of the process so that the structure of the tool was in line with the needs of the government and that research findings were more likely to be relevant to and used by policy-makers.^17^ Due to this process, the policy brief may avoid being too “controversial,” supporting policy-makers in incrementally changing and improving policies. This is different from research-driven approaches that are independent from government, which aim to build up public pressure on policy-makers to increase efforts, eg, by attracting media attention.^9^

 Fourth, the approach has the potential to be turned into regular monitoring, as the government can use target group specific policy briefs to maintain an overview of the current status of PA promotion and to track progress over time. As the development of these policy briefs is based on a rapid process (six months), resources are manageable and well invested to inform policy-making in the field of PA promotion.

 Fifth, the tool is adaptable to the needs of policy-makers and, importantly, to the already existing evidence-base of the health effects of PA for the target group. Policy-makers might ask to focus on specific aspects. For some target groups, recommendations on PA and PA promotion are firmly in place, and little additional research may be needed. For other target groups (eg, people with specific chronic health conditions), more systematic reviews of the existing knowledge base might be warranted.

 Finally, the tool itself is potentially transferable to other nations and target groups. The transferability to other target groups was already a requirement during tool development, and was successfully tested in Germany for adults, older adults, and adults with non-communicable diseases.^39^ The transferability to other countries is not guaranteed, as the context and involved institutions may call for different foci, indicators, and structure. However, transferability is supported by the international focus of the tool, in particular by its reference to WHO’s PA guidelines (PA recommendations) and international recommendations for PA promotion issued by WHO, the Council of the EU, and the International Society for Physical Activity and Health. Furthermore, the tool is not adapted to country-specific aspects of Germany such as the federal system, ie, it can easily be applied to monitor PA promotion at national level in other countries.

 In comparison to existing tools that monitor PA promotion, the TARGET:PA tool has a rather broad focus. As it includes information from PA prevalence rates to PA policies, it seems to be most similar to the Report Cards of the Global Observatory for PA^14^ and the Active Healthy Kids Global Alliance^15,36^ – while tools such as the HEPA PAT^16^ and the PA Environment Policy Index^13^ focus exclusively on policies. This raises the question whether the development of a new tool is actually needed: With plenty of tools to monitor PA promotion readily available, an integration of existing tools might have more benefits than the development of new ones. On the other hand, in our specific case, existing tools simply did not fulfil the needs expressed by German government officials. A way forward might be to explore whether countries that already apply other monitoring instruments could use the methodology of the TARGET:PA tool to inform the further development of these tools to better integrate good and routine practice into the monitoring of PA promotion (rather than substituting an entirely new tool for the ones currently in use).

 The goal of this study has been to report our experience of developing a PA promotion monitoring tool at the request of policy-makers and tailored to their needs. Consequently, information on the development process, the tool itself, and the resulting policy brief and background document is reported exclusively from the perspective of the participating researchers. This implies that there was no way for us to clean data, address data gaps, reduce reporting bias, or provide others with access to the raw data. The results reported in this paper have to be interpreted against this exploratory backdrop. Nonetheless, our experience may be useful to other researchers faced with similar demands for scientific support from their own governments or other institutions.

 Another potential limitation is that, while the individual elements of the tool can be considered to be evidence-based, the process of developing TARGET:PA was not theory driven but rather primarily focused on the needs of government officials. Furthermore, it might be difficult to collect data on routine practice as the quality of the results depends on the level of expertise of selected experts and the number of experts involved. Likewise, the transferability to other nations has not yet been tested, and it needs to be investigated whether the tool fits to other political cultures and country contexts. A particular challenge is to ensure not to exclude or under-represent PA promotion interventions and policies like infrastructures for walking and cycling, which may benefit the population in question but are usually labelled as being directed at the “general population.” In addition, important aspects such as diversity, social inequalities and the specific needs of socially disadvantaged groups are not addressed systematically; however, the tool can be applied with this particular focus (ie, by showcasing gender-specific prevalence rates, or by assessing the impact of good practice projects on health equity). Nevertheless, the innovative aspects seem to outweigh potential limitations, and future initiatives may help to further develop the tool.

## Conclusions

 The TARGET:PA tool provides added value for the monitoring of PA promotion as it has a unique focus on routine practice, assesses good practice projects objectively and identifies policy gaps systematically. It was tested for the target group of children and adolescents in 2021/2022^22^ and susequently served as a conceptual basis for the development of three additional PA policy briefs requested by the German government. While developed to specifically suit the German context, it has the potential to be adapted to other countries. This may be achieved either by taking inspiration from the collaborative development process or—where appropriate—by directly using the tool to collect information on PA promotion in other countries or contexts.

## Ethical issues

 Ethical approval did not apply. No data pertaining to individuals were used to report on the tool development process.

## Conflicts of interest

 Authors declare that they have no conflicts of interest.
